# Assessment of dietary patterns in celiac disease patients using factor analysis method and their relationship with dietary intakes and body mass index

**DOI:** 10.1186/s40795-024-00849-7

**Published:** 2024-03-06

**Authors:** Mehrnaz Morvaridi, Narges Sadeghi, Pezhman Alavinejad, Mehdi Sadeghian, Negin Tahvilian, Hossein Bavi Behbahani, Sara Ebrahimi, Farnaz Farsi

**Affiliations:** 1 Department of Nutrition Sciences, School of Public Health, Iran University of Medical Sciences, Tehran, Iran; 2 Department of Nutrition, School of Allied Medical Science, Ahvaz Jundishapur University of Medical Sciences, Ahvaz, Iran; 3 Nutrition and Metabolic Diseases Research Center, Ahvaz Jundishapur University of Medical Sciences, Ahvaz, Iran; 4 Alimentary Tract Research Center, Ahvaz Jundishapur University of Medical Sciences, Ahvaz, Iran; 5 Ph.D. of Nutrition, YAS Hospital, Tehran University of Medical Sciences, Associate faculty, Tehran, Iran; 6 Department of Nutrition, School of Public Health, Shahid Sadoughi University of Medical Sciences, Yazd, Iran; 7 Nutrition and Food Security Research Center, Shahid Sadoughi University of Medical Sciences, Yazd, Iran; 8 Student Research Committee, Ahvaz Jundishapur University of Medical Sciences, Ahvaz, Iran; 9 Institute for Physical Activity and Nutrition (IPAN), School of Exercise and Nutrition Sciences, Deakin University, Geelong, VIC 3220 Australia; 10 Minimally Invasive Surgery Research Center, Iran University of Medical Sciences, Tehran, Iran

**Keywords:** Celiac disease, Dietary patterns, Dietary intakes

## Abstract

**Background/Objectives:**

Celiac disease (CD) is a systemic and autoimmune enteropathy of the gastrointestinal tract with malabsorption characteristics. The only effective treatment for CD is adhere strictly to a gluten-free diet (GFD) throughout life. We evaluated the dietary patterns in celiac disease patients and their association with dietary intakes and anthropometric measurements in Iran.

**Subjects/Methods:**

This is a case-control study on 182 participants who were referred to the Khuzestan Celiac Association, Iran. Nutritional information was collected using a validated 147-item semi-quantitative food frequency questionnaire (FFQ). The software Stata (StataCorp, Version 14.0) was used to analyze the data. Principal component analysis (PCA) was used to obtain participants’ dietary patterns.

**Results:**

A significant relationship was observed between age and body mass index (BMI) across quartiles of the healthy dietary pattern score (*P* < 0.001, *P* = 0.001, and *P* = 0.001, respectively), indicating that as age and BMI increased, participants demonstrated greater adherence to the healthy dietary pattern. Individuals with the highest adherence to the healthy dietary pattern had the lowest odds ratio for celiac disease (CD) (Q1: reference; Q2: 1.96, 95% CI: 0.84–4.55; Q3: 0.61, 95% CI: 0.27–1.42; Q4: 0.10, 95% CI: 0.03–0.33, P trend < 0.001), and this association remained significant after adjusting for BMI (adjusted P trend = 0.003) and energy intake (adjusted P trend < 0.001). Moreover, there was a significant association between the lowest odds ratio for CD and the highest adherence to the unhealthy dietary pattern after adjustment for energy intake (Q1: reference; Q2: 0.38, 95% CI: 0.13–1.12; Q3: 0.21, 95% CI: 0.06–0.71; Q4: 0.07, 95% CI: 0.02–0.29, adjusted P trend < 0.001). Additionally, a significant association was observed between the odds ratio for CD and the mixed dietary pattern score (Q1: reference; Q2: 6.01, 95% CI: 2.29–15.72; Q3: 2.47, 95% CI: 0.93–6.55; Q4: 4.84, 95% CI: 1.84–12.66, P trend = 0.02), and this association remained significant after adjustment for energy intake (adjusted P trend < 0.001).

**Conclusions:**

The findings of the present study indicate that individuals who adhere to healthy dietary patterns have a lower incidence of celiac disease.

**Supplementary Information:**

The online version contains supplementary material available at 10.1186/s40795-024-00849-7.

## Introduction

Celiac disease (CD) is a systemic and autoimmune enteropathy of the gastrointestinal tract with malabsorption characteristics triggered by consuming gluten-containing foods including wheat, barley, rye and other similarly related grains in genetically susceptible individuals [1–3]. Clinically, manifestations of CD can vary from patient to patient, mainly including diarrhea, constipation, abdominal cramps, bloating, fatigue, anorexia, weight loss, unusual skin rash, osteoporosis, anemia, infertility, late puberty, and short stature [4, 5]. In recent years, the prevalence of CD has increased and approximately 1% of the world’s population is suffering from this disorder. Its overall prevalence in the Iranian population has been reported from 2 to 3% respectively on the basis of biopsy and serological test for diagnosis, indicating the need for more attention to this disease and timely diagnosis CD in Iran [3, 6].

Strict adherence to a gluten-free diet (GFD) is the sole effective treatment for celiac disease (CD), leading to recovery of CD-related manifestations and normalization of histological and laboratory findings [7, 8]. Despite the belief that a strict GFD cannot lead to nutritional deficiencies, evidence suggests potential nutritional imbalances [2]. Recent studies recommend monitoring CD patients on a GFD for nutritional deficiencies, particularly in micro- and macro-nutrients and fiber intake [2, 7, 9]. Controversies persist regarding the nutritional adequacy of the GFD, sparking ongoing debates. The challenges faced by CD patients in implementing the diet are highlighted due to limitations, inability to consume a variety of foods containing gluten, and differences in the composition of gluten-free substitutes [2, 10, 11]. Researchers highlight the importance of nutritional assessment based on dietary patterns to understand their connection with diseases. Nutritionists emphasize the evaluation of overall dietary patterns over individual foods or nutrients. Analyzing food intakes through a dietary pattern offers a comprehensive approach to disease prevention and treatment, with significant implications for public health [7, 10, 12].

Anthropometric measures are crucial for understanding human metabolism in a variety of medical disorders, including celiac disease. These metrics include weight, body mass index, skinfold thickness, fat mass, and bone mineral density. Previous studies showed adolescent boys with CD were leaner and girls with CD were shorter compared with the general population. However, the clinical relevance of the small differences suggests that when CD is diagnosed during childhood, final weight and height are not severely impaired [13, 14]. Therefore, Celiac disease significantly changes anthropometric measurements, and adherence to a gluten-free diet has the potential to improve these metrics. However, anthropometric measures in individuals with celiac disease may not attain levels comparable to those observed in healthy individuals [15].

Given that no large study has been conducted to examine the dietary patterns of patients in Iran, there are no large studies on adults regarding the nutritional adequacy of the GFD. Therefore, we evaluated the dietary patterns in celiac disease patients using the factor analysis method and their association with dietary intakes and anthropometric measurements in Khuzestan province; which can provide a clear picture of deficiencies and common malnutrition in patients and provide a basis for improving education and improving the quality of diets.

## Materials and methods

### Study population

This case-control study was conducted on participants referred to the Khuzestan Celiac Association in Ahvaz, Iran, between April and August of 2021. Celiac disease patients in the case group were selected using information from the Celiac Association and a diagnosis made by a gastroenterologist. Histological findings, serological testing (including IgA anti-transglutaminase antibody levels), the Marsh grading system for categorizing of lesions associated with celiac disease, and the patient’s reaction to a gluten-free diet were used to determine the diagnosis criteria. The inclusion criteria for the case group comprised individuals older than 18 years of age who adhered to a gluten-free diet. The exclusion criteria were as follows: diagnosis of celiac disease less than 2 years ago; age less than 18 years or more than 70 years; the presence of metabolic and chronic diseases (such as diabetes, Crohn’s disease, cardiovascular diseases, neurological diseases, cancer, neurodegenerative diseases, and rheumatoid arthritis); pregnancy or breastfeeding; and being on special diets. The control group consisted of people who were selected from a healthy population and ranged in age from 18 to 70. The criteria for excluding individuals from the control group were: age below 18 or above 70 years; the presence of metabolic and chronic conditions; pregnancy or breastfeeding; and adherence to specialized diets. Convenience sampling was used to choose participants, and during the allotted time, healthy individuals referred to the Khuzestan Celiac Association and those diagnosed with celiac disease were evaluated in accordance with the inclusion criteria.

### Measurements

A questionnaire, including socioeconomic and demographic information such as age, gender, marital status, race, access to gluten-free food products, and history of other diseases, was completed by the participants. Anthropometric assessments of standing height without shoes, and standing weight with minimum clothing were measured and body mass index (BMI; kg/m2) was calculated by dividing the weight by the height squared. Waist circumference was measured using a tape measure at the midpoint between the last rib and the iliac crest.

Nutritional information was collected using a validated 147-item semi-quantitative food frequency questionnaire (FFQ) [16] in collaboration with a trained professional interviewer who taught patients how to fill out questionnaires correctly. To fill out the questionnaires, patients were instructed on the portion sizes of each food item by a set of household units (e.g., tablespoon, cup, small bowl, teaspoon plate, glass, and spatula) and a validated food album [17]. The 147-item semi-quantitative FFQ assessed the patient’s dietary intakes of a given serving of each food item in 4 frequency of consumption categories (daily, weekly, monthly, and yearly).

### Data analysis

The study utilized Stata software (StataCorp, Version 14.0) for data analysis, employing descriptive statistics such as mean ± standard deviation for quantitative variables and frequency/percentage for qualitative variables. Principal component analysis (PCA) was employed to discern dietary patterns from FFQ data. Thirty-six food groups were categorized through PCA (Supplementary Table 1), considering factor loadings above 0.20 as significant contributors to dietary patterns (Supplementary Table 2) [18, 19]. The number of key dietary patterns to retain was determined based on scree plot analysis (factors with eigenvalues > 1.5) and the interpretability of the identified patterns (Supplementary Fig. 1). The analysis identified three categories: I (healthy), II (unhealthy), and III (mixed) patterns. The healthy pattern exhibited a higher intake of nutritious foods, while the unhealthy pattern showed a preference for less healthy options. The mixed pattern displayed a blend of both. ANOVA and chi-square methods were used to compare quantitative and qualitative variables, respectively, between the quartiles of dietary pattern score. Also, ANOVA tests were used to adjust variables for BMI (model 1) and energy intake (model 2).

## Results

**Fig. 1:**
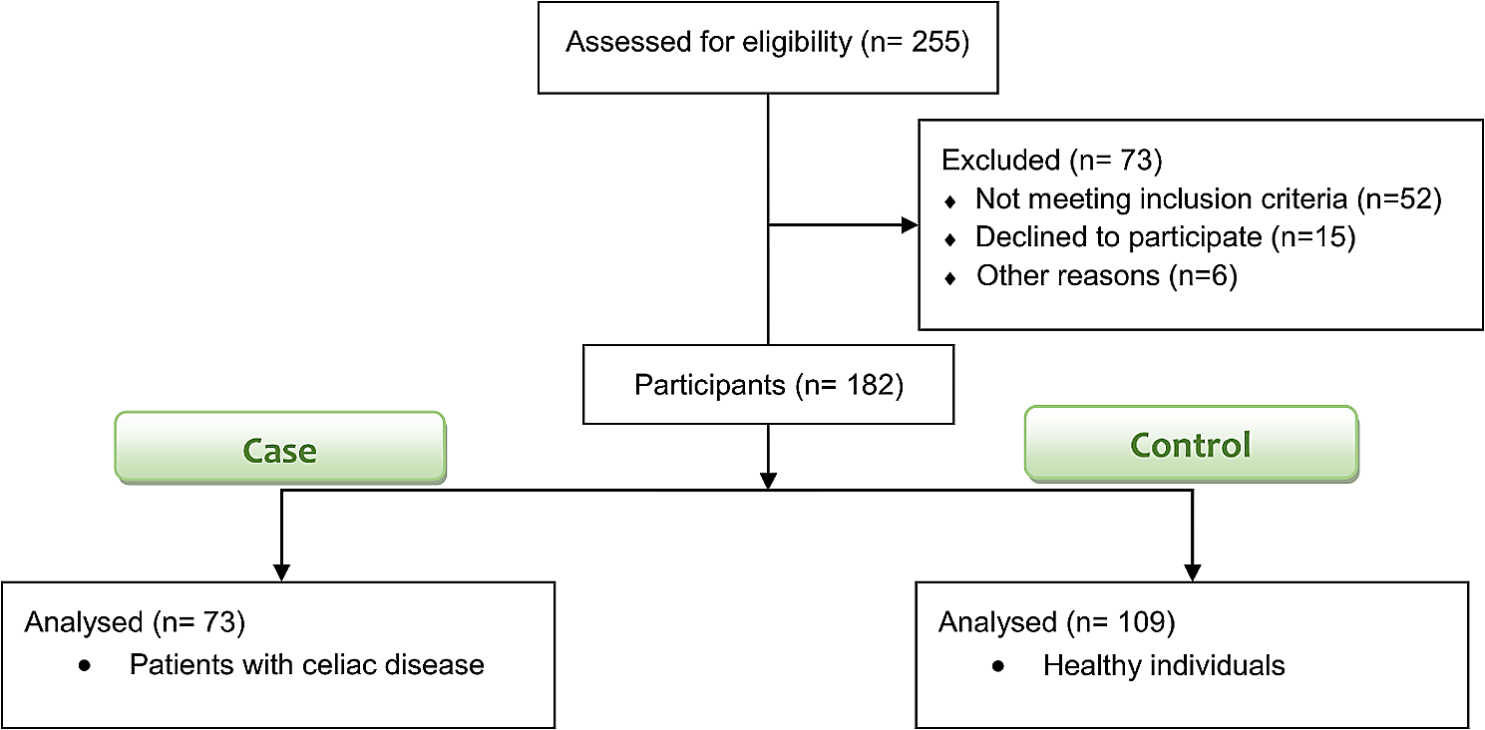
Study flowchart

Of 255 individuals who participated in the study, 182 met the inclusion criteria (Fig. 1). Demographic characteristics and dietary intakes of cases and control participants are provided in Table 1. Individuals diagnosed with celiac disease were more likely to be female, younger, and single. They also had a lower BMI and were less educated than the control group. Table 1 depicts that individuals with celiac disease had a lower intake of most macronutrients, with the exception of trans fatty acids. Additionally, Table 1 presents a comparison of micronutrient intake between patients with celiac disease and the control group.

**Table 1:** Demographic characteristics and dietary intakes of cases and controls

Variable	Groups		
	Controls (*n* = 109)	Cases (*n* = 73)	P*
Age (years)	42.39 ± 11.52	28.95 ± 17.35	< 0.001
BMI (kg/m^2^)	29.05 ± 4.27	22.45 ± 6.41	< 0.001
Female (%)	50	73	0.02
Married (%)	83	59	< 0.001
Educated (%)	98	88	< 0.001
Total energy (kcal/day)	3339.68 ± 1304.29	2251.17 ± 662.62	< 0.001
Carbohydrate (g/day)	495.38 ± 217.19	338.20 ± 109.87	< 0.001
Protein (g/day)	118.27 ± 44.38	65.72 ± 23.81	< 0.001
Total fat (g/day)	106.45 ± 42.15	74.76 ± 24.73	< 0.001
Dietary fiber (g/day)	42.56 ± 20.62	22.91 ± 9.58	< 0.001
SFA (g/day)	34.05 ± 17.38	23.71 ± 8.64	< 0.001
MUFA (g/day)	33.73 ± 17.44	19.26 ± 6.79	< 0.001
PUFA (g/day)	26.78 ± 15.18	11.80 ± 10.72	< 0.001
TFA (g/day)	12.98 ± 12.88	229.67 ± 126.35	< 0.001
Cholesterol (mg/day)	322.87 ± 189.54	186.62 ± 99.14	< 0.001
Vitamin C (mg/day)	245.82 ± 129.11	21.29 ± 0.86	< 0.001
Vitamin B1 (mg/day)	3.78 ± 1.71	1.67 ± 0.79	< 0.001
Vitamin B2 (mg/day)	2.72 ± 1.16	32.74 ± 15.42	< 0.001
Vitamin B3 (mg/day)	98.21 ± 75.04	5.73 ± 2.14	< 0.001
Vitamin B5 (mg/day)	9.64 ± 3.89	2.58 ± 1.14	< 0.001
Vitamin B6 (mg/day)	3.91 ± 1.57	425.08 ± 139.05	< 0.001
Vitamin B9 (µg/day)	649.94 ± 249.79	351.01 ± 112.21	< 0.001
Vitamin B12 (µg/day)	5.56 ± 5.42	774.17 ± 388.92	< 0.001
Vitamin A (mg/day)	1257.46 ± 913.52	5599.74 ± 3077.70	< 0.001
Bata Carotene (mg/day)	8794.23 ± 6562.59	752.42 ± 374.66	< 0.001
Calcium (mg/day)	1149.89 ± 468.19	14.30 ± 5.08	< 0.001
Iron (mg/day)	26.47 ± 11.69	332.82 ± 132.97	< 0.001
Magnesium (mg/day)	645.53 ± 279.59	1154.35 ± 446.03	0.124
Phosphor (mg/day)	2176.07 ± 914.71	3602.56 ± 1514.07	0.061
Potassium (mg/day)	5994.68 ± 2608.65	1867.49 ± 798.34	< 0.001
Sodium (mg/day)	5113.71 ± 2374.95	9.96 ± 3.45	< 0.001
Zinc (mg/day)	18.45 ± 7.96	2.01 ± 0.94	< 0.001
Copper (mg/day)	4.22 ± 2.52	5.17 ± 1.76	0.046
Manganese (mg/day)	10.23 ± 5.16	59.19 ± 19.53	< 0.001
Selenium (mg/day)	93.46 ± 30.93	77.53 ± 30.46	0.95

All values are mean ± SD or percent

*Obtained from independent sample t test or Chi-square test, where appropriate

Abbreviatios: SFA, saturated fatty acid; MUFA, monounsaturated fatty acid; PUFA, polyunsaturated fatty acid; TFA, trans fatty acid

Table 2 presents the characteristics and dietary intakes of participants across healthy, unhealthy, and mixed dietary patterns. There was a significant relationship between age and BMI with quartiles of healthy dietary pattern score (*P* < 0.001, *P* = 0.001, and *P* = 0.001, respectively). With increasing age and BMI, participants had the highest adherence to the healthy dietary pattern. Furthermore, Table 2 delineates the association between the participants’ intake of macronutrients and micronutrients across the first to fourth quartiles of the dietary pattern scores.

**Table 2:** Characteristics of study participants by quartiles of pattern scores^1^

Variable	Quartiles of healthy dietary pattern score					Quartiles of unhealthy dietary pattern score					Quartiles of mixed dietary pattern score				
	1 (Lowest)	2	3	4 (Highest)	P^3^	1 (Lowest)	2	3	4 (Highest)	P^3^	1 (Lowest)	2	3	4 (Highest)	P^3^
Participants^2^ (n)	45	46	46	45		45	46	46	45		45	46	46	45	
Age^2^ (year)	32.13 ± 16.66	31.26 ± 15.19	40.15 ± 12.47	44.51 ± 12.47	< 0.001	36.29 ± 16.24	41.35 ± 13.97	33.37 ± 16.09	36.98 ± 15.37	0.10	41.87 ± 13.76	36.65 ± 16.32	34.61 ± 14.11	34.93 ± 17.27	0.097
BMI^2^ (kg/m^2)^	25.29 ± 7.48	24.02 ± 5.71	27.47 ± 5.25	28.87 ± 4.79	0.001	25.94 ± 6.42	26.20 ± 5.67	25.56 ± 5.87	27.94 ± 6.51	0.26	27.91 ± 5.19	25.45 ± 6.16	27.12 ± 5.61	25.15 ± 7.19	0.95
Male^2^, %	44	35	41	44	0.76	47	30	41	47	0.35	31	46	46	42	0.45
Married^2^, %	78	67	67	82	0.25	84	65	72	73	0.20	76	70	80	69	0.49
Total energy (kcal/day)	3318.82 ± 1335.58	2435.71 ± 923.49	2699.53 ± 1062.57	3173.17 ± 1318.19	0.001	3605.16 ± 1226.17	2606.22 ± 594.58	2546.92 ± 945.92	2868.53 ± 1600.35	< 0.001	3067.80 ± 1584.64	2494.65 ± 1315.62	2917.11 ± 840.42	3141.52 ± 898.46	0.05
Protein (g/day)	99.46 ± 45.34	76.59 ± 35.33	91.30 ± 40.84	121.99 ± 48.53	< 0.001	113.26 ± 45.37	87.16 ± 31.56	88.47 ± 40.66	100.30 ± 57.27	0.019	104.66 ± 53.63	83.69 ± 49.27	97.76 ± 31.74	102.94 ± 42.86	0.111
Carbohydrate (g/day)	495.76 ± 221.40	368.61 ± 151.51	407.39 ± 170.93	460.19 ± 218.60	0.10	539.84 ± 210.03	395.23 ± 85.36	388.97 ± 163.86	407.73 ± 255.15	< 0.001	471.09 ± 257.49	367.41 ± 209.52	418.19 ± 132.83	475.05 ± 150.68	0.027
Total fat (g/day)	110.69 ± 48.55	77.79 ± 28.88	85.40 ± 33.53	101.61 ± 36.11	< 0.001	119.03 ± 36.56	82.35 ± 27.32	76.47 ± 24.95	97.74 ± 50.05	< 0.001	92.26 ± 52.88	81.27 ± 35.52	101.13 ± 33.78	97.39 ± 29.24	0.067
Dietary fiber (g/day)	33.74 ± 15.93	24.81 ± 14.08	26.75 ± 14.39	43.59 ± 25.99	0.001	41.11 ± 18.13	20.08 ± 6.47	27.07 ± 13.83	34.62 ± 21.39	< 0.001	34.62 ± 24.30	24.10 ± 17.69	29.19 ± 10.72	35.62 ± 16.64	0.031
SFA (g/day)	33.95 ± 14.73	22.63 ± 7.78	24.46 ± 10.39	30.54 ± 20.17	0.001	37.22 ± 13.52	20.67 ± 5.48	23.28 ± 9.01	30.62 ± 17.25	< 0.001	27.44 ± 18.91	23.02 ± 14.11	28.96 ± 12.44	31.38 ± 10.09	0.076
MUFA (g/day)	30.34 ± 14.81	20.03 ± 8.55	22.68 ± 14.09	27.62 ± 18.03	0.007	32.48 ± 18.06	18.99 ± 5.61	21.03 ± 9.07	27.94 ± 16.19	0.001	27.78 ± 21.32	21.06 ± 12.41	26.76 ± 12.18	25.77 ± 11.59	0.268
PUFA (g/day)	15.95 ± 21.36	4.24 ± 9.22	10.18 ± 15.76	21.41 ± 18.76	0.002	14.24 ± 21.52	2.07 ± 5.00	8.88 ± 12.14	19.95 ± 20.89	< 0.001	16.78 ± 20.99	5.59 ± 12.36	13.39 ± 17.30	13.29 ± 18.92	0.097
TFA (g/day)	156.41 ± 191.07	167.63 ± 101.44	139.02 ± 132.12	61.84 ± 88.99	0.077	225.11 ± 224.85	117.03 ± 82.98	133.59 ± 111.09	66.79 ± 88.38	< 0.001	66.54 ± 77.03	138.10 ± 79.13	140.89 ± 127.89	188.76 ± 202.08	0.018
Cholesterol (mg/day)	242.43 ± 155.81	177.47 ± 74.27	272.48 ± 152.38	326.49 ± 237.53	0.005	276.00 ± 128.17	211.83 ± 116.76	198.33 ± 127.49	278.25 ± 208.57	0.079	184.57 ± 136.95	170.89 ± 165.71	217.84 ± 83.95	348.99 ± 147.42	< 0.001
Vitamin C (mg/day)	104.27 ± 144.03	44.86 ± 110.37	88.53 ± 117.16	228.89 ± 180.95	< 0.001	113.32 ± 180.93	36.35 ± 99.06	74.13 ± 102.19	160.80 ± 157.41	0.004	125.00 ± 147.10	52.56 ± 117.21	104.33 ± 131.35	123.47 ± 168.30	0.157
Vitamin B1 (mg/day)	2.89 ± 1.69	1.86 ± 1.15	2.13 ± 1.07	3.71 ± 2.17	< 0.001	3.19 ± 1.83	1.63 ± 0.59	2.23 ± 1.32	2.95 ± 1.88	0.001	2.60 ± 2.00	1.89 ± 1.46	2.35 ± 1.09	3.14 ± 1.65	0.009
Vitamin B2 (mg/day)	20.55 ± 20.09	25.93 ± 16.63	23.07 ± 21.51	5.67 ± 5.00	0.002	27.90 ± 23.33	32.02 ± 18.48	17.66 ± 13.76	10.15 ± 13.32	< 0.001	15.71 ± 22.19	21.59 ± 14.84	20.57 ± 19.61	22.69 ± 20.3	0.587
Vitamin B3 (mg/day)	42.46 ± 54.28	21.35 ± 42.03	43.79 ± 75.58	89.18 ± 91.87	0.005	64.53 ± 92.60	13.42 ± 22.59	45.13 ± 60.12	46.38 ± 63.15	0.040	85.96 ± 98.36	30.32 ± 68.92	32.51 ± 39.22	38.00 ± 47.19	0.009
Vitamin B5 (mg/day)	5.98 ± 4.45	3.51 ± 2.79	4.69 ± 3.57	9.41 ± 5.30	< 0.001	6.08 ± 4.70	2.96 ± 1.92	4.82 ± 3.82	7.27 ± 4.92	0.001	6.12 ± 5.42	3.69 ± 3.87	5.35 ± 3.63	6.54 ± 4.25	0.036
Vitamin B6 (mg/day)	262.59 ± 261.54	329.39 ± 205.14	250.03 ± 219.71	90.02 ± 165.24	0.005	328.83 ± 265.69	413.38 ± 193.12	226.04 ± 185.11	117.76 ± 19.07	< 0.001	123.81 ± 165.75	281.04 ± 170.19	238.89 ± 232.45	318.47 ± 283.56	0.016
Vitamin B9 (µg/day)	303.22 ± 376.61	129.37 ± 279.53	220.20 ± 298.76	528.56 ± 398.13	0.001	292.19 ± 429.96	68.10 ± 187.52	225.24 ± 317.57	417.79 ± 359.23	0.001	364.70 ± 368.55	140.79 ± 302.67	270.19 ± 335.93	305.79 ± 386.46	0.101
Vitamin B12 (µg/day)	472.71 ± 580.48	564.07 ± 388.91	484.41 ± 453.56	202.63 ± 397.32	0.083	666.88 ± 586.33	673.35 ± 406.71	385.33 ± 343.49	245.56 ± 461.42	< 0.001	148.31 ± 191.83	441.51 ± 295.03	436.87 ± 407.29	677.78 ± 651.59	0.001
Vitamin A (mg/day)	3620.99 ± 3666.24	4180.46 ± 2431.23	4351.88 ± 3846.72	2883.94 ± 2613.82	0.435	5047.66 ± 3446.99	5135.81 ± 3257.06	3148.88 ± 2437.51	2737.89 ± 3273.53	0.003	1654.20 ± 1209.90	3490.39 ± 2102.49	3608.41 ± 2599.23	5534.14 ± 4283.29	< 0.001
Bata Carotene (mg/day)	3811.43 ± 4960.10	2068.69 ± 3858.19	2935.97 ± 3467.14	10272.83 ± 9060.58	< 0.001	4404.27 ± 5799.50	1508.84 ± 2519.83	2960.57 ± 4488.92	6428.99 ± 7356.66	0.005	4515.37 ± 6516.96	2513.92 ± 4518.64	3715.76 ± 4289.19	5136.02 ± 6929.09	0.264
Calcium (mg/day)	534.35 ± 660.53	222.93 ± 498.08	370.51 ± 484.04	1023.08 ± 714.09	< 0.001	498.43 ± 738.42	118.40 ± 307.92	426.80 ± 582.59	745.56 ± 656.80	0.001	639.77 ± 653.71	270.54 ± 582.78	487.08 ± 550.41	535.92 ± 694.71	0.154
Iron (mg/day)	216.97 ± 229.62	252.47 ± 135.58	213.28 ± 164.74	94.19 ± 133.11	0.028	267.75 ± 191.67	305.63 ± 130.68	182.56 ± 130.44	124.39 ± 207.74	< 0.001	114.83 ± 124.89	213.31 ± 110.29	199.93 ± 174.99	265.24 ± 238.41	0.023
Magnesium (mg/day)	1040.05 ± 637.09	970.72 ± 333.33	872.55 ± 327.32	820.49 ± 366.69	0.293	1202.33 ± 441.98	1052.02 ± 294.56	853.04 ± 272.51	785.61 ± 608.16	0.001	758.12 ± 349.06	877.49 ± 262.15	904.98 ± 378.45	1148.11 ± 617.39	0.006
Phosphor (mg/day)	3188.08 ± 1992.18	2934.73 ± 1014.92	3062.02 ± 1275.51	2809.95 ± 1296.58	0.805	3822.51 ± 1420.16	3281.28 ± 1116.44	2695.02 ± 824.87	2591.23 ± 1942.93	0.004	2235.86 ± 1019.47	2628.29 ± 779.09	2911.23 ± 1060.44	3884.65 ± 1958.21	< 0.001
Potassium (mg/day)	3919.48 ± 2763.88	2406.48 ± 1976.66	3166.13 ± 2114.56	5623.32 ± 3369.62	< 0.001	4121.18 ± 3030.22	2113.48 ± 1255.83	3072.89 ± 2230.14	4541.30 ± 3087.60	0.002	4370.79 ± 3420.41	2476.19 ± 2480.59	3471.47 ± 1926.62	3997.25 ± 2700.02	0.037
Sodium (mg/day)	2416.43 ± 3158.25	864.48 ± 1918.38	1994.35 ± 2902.06	3930.25 ± 3240.71	0.003	2517.44 ± 3866.75	531.26 ± 1489.57	1985.97 ± 2719.69	2903.05 ± 2772.99	0.012	3524.30 ± 3662.03	1317.66 ± 2950.71	1950.28 ± 2428.94	1980.70 ± 2608.92	0.056
Zinc (mg/day)	9.55 ± 9.14	4.76 ± 7.08	7.77 ± 9.01	16.15 ± 11.75	< 0.001	10.44 ± 11.97	3.48 ± 4.37	8.19 ± 8.98	11.38 ± 9.55	0.008	12.57 ± 12.07	5.37 ± 9.39	8.13 ± 7.68	9.57 ± 8.78	0.047
Copper (mg/day)	4.71 ± 2.09	4.66 ± 1.75	4.89 ± 2.09	5.06 ± 3.08	0.918	6.11 ± 2.32	5.40 ± 1.39	4.11 ± 1.42	4.02 ± 2.47	< 0.001	4.67 ± 2.58	4.36 ± 1.97	4.58 ± 2.03	5.35 ± 2.06	0.229
Manganese (mg/day)	42.41 ± 34.65	48.33 ± 24.90	35.33 ± 23.36	20.19 ± 18.37	0.005	52.92 ± 32.95	54.71 ± 21.11	36.23 ± 22.82	21.86 ± 24.83	< 0.001	24.87 ± 19.24	41.44 ± 19.24	37.37 ± 29.40	47.27 ± 35.82	0.032
Selenium (mg/day)	88.89 ± 29.16	78.73 ± 31.07	81.11 ± 31.69	88.22 ± 37.58	0.473	92.17 ± 32.99	78.16 ± 24.09	82.09 ± 31.12	83.87 ± 35.35	0.434	79.39 ± 38.67	70.58 ± 25.90	87.22 ± 25.19	95.22 ± 32.19	0.007

1 Participants in the first and fourth Quartiles had the lowest and highest adherence to the dietary pattern, respectively

2 Data are means ± SD unless indicated

3 from ANOVA for quantitative variables and chi-square for qualitative variables

Abbreviatios: SFA, saturated fatty acid; MUFA, monounsaturated fatty acid; PUFA, polyunsaturated fatty acid; TFA, trans fatty acid

The association of dietary patterns scores quartiles with CD is shown in Table 3. The lowest odd ratio for CD was seen in the highest adherence to the healthy dietary pattern (Q1: reference; Q2: 1.96, 95% CI: 0.84–4.55; Q3: 0.61, 95% CI: 0.27–1.42; Q4: 0.10, 95% CI: 0.03–0.33, P trend < 0.001) and the association remained significant after adjustment for BMI (model 1) (Q1: reference; Q2: 1.88, 95% CI: 0.69–5.08; Q3: 0.96, 95% CI: 0.36–2.59; Q4: 0.16, 95% CI: 0.04–0.58, adjusted P trend = 0.003) and energy intake (model 2) (Q1: reference; Q2: 0.68, 95% CI: 0.24–1.97; Q3: 0.23, 95% CI: 0.08–0.65; Q4: 0.04, 95% CI: 0.98–0.99, adjusted P trend < 0.001). Also, there was a significant association between the lowest odd ratio for CD and the highest adherence to the unhealthy dietary pattern after adjustment for energy intake (Q1: reference; Q2: 0.38, 95% CI: 0.13–1.12; Q3: 0.21, 95% CI: 0.06–0.71; Q4: 0.07, 95% CI: 0.02–0.29, adjusted P trend < 0.001). Moreover, there was a significant association of odd ratio for celiac disease and mixed dietary pattern score (Q1: reference; Q2: 6.01, 95% CI: 2.29–15.72; Q3: 2.47, 95% CI: 0.93–6.55; Q4: 4.84, 95% CI: 1.84–12.66, P trend = 0.02) and the association remained significant after adjustment for energy intake (Q1: reference; Q2: 6.82, 95% CI: 2.13–21.89; Q3: 5.90, 95% CI: 1.73–20.12; Q4: 22.76, 95% CI: 5.95–87.02, adjusted P trend < 0.001). However, the odds ratio for celiac disease was not associated with the unhealthy dietary pattern score and mixed dietary pattern score after adjusting for BMI (P trend = 0.25, adjusted P trend = 0.51 and adjusted P trend = 0.06, respectively).

**Table 3:** Multivariate odds ratios for celiac disease across tertiles of dietary pattern scores^1,2^

	Quartiles of healthy pattern score					Quartiles of unhealthy dietary pattern score					Quartiles of mixed dietary pattern score				
	1 (Lowest)	2	3	4 (Highest)	P-trend	1 (Lowest)	2	3	4 (Highest)	P-trend	1 (Lowest)	2	3	4 (Highest)	P-trend
Celiac disease															
Crude	1.00	1.96 (0.84–4.55)	0.61 (0.27–1.42)	0.10 (0.03–0.33)	< 0.001	1.00	1.65 (0.72–3.79)	1.38 (0.59–3.19)	0.59 (0.25–1.47)	0.25	1.00	6.01 (2.29–15.72)	2.47 (0.93–6.55)	4.84 (1.84–12.66)	0.02
Model 1^3^	1.00	1.88 (0.69–5.08)	0.96 (0.36–2.59)	0.16 (0.04–0.58)	0.003	1.00	2.09 (0.78–5.62)	1.49 (0.55–4.01)	0.73 (0.25–2.15)	0.51	1.00	5.39 (1.82–15.94)	2.72 (0.9–8.19)	4.01 (1.33–12.11)	0.06
Model 2^4^	1.00	0.68 (0.24–1.97)	0.23 (0.08–0.65)	0.04 (0.98–0.99)	< 0.001	1.00	0.38 (0.13–1.12)	0.21 (0.06–0.71)	0.07 (0.02–0.29)	< 0.001	1.00	6.82 (2.13–21.89)	5.90 (1.73–20.12)	22.76 (5.95–87.02)	< 0.001

^1^participants in the first and forth quartiles had the lowest and highest adherence to the dietary pattern, respectively

^2^Values are OR (95% CI)

^3^Adjusted for body mass index

^4^Adjusted for energy intake

## Discussion

In this case-control study based on the population, differences in dietary nutrient intake were evident between the case and control groups. It was observed that participants’ adherence to a healthy diet increased with higher age, BMI, and total energy intake. Notably, a significant association was found between total energy intake and quartiles of unhealthy and mixed dietary pattern scores. Surprisingly, the study discovered that the highest adherence to both healthy and unhealthy dietary patterns was linked to a reduced likelihood of having celiac disease, even after adjusting for BMI and/or energy intake. Additionally, a significant inverse correlation was identified between the odds ratio of CD incidence and the mixed dietary pattern score, even after adjusting for energy intake. Generally, little information exists available on the dietary pattern of CD in the GF diet and whether differences in macro-nutrient, micronutrient intake or dietary quality may be seen in celiac individuals of different ethnicities [20, 21]. To the best of our knowledge, no study has investigated the dietary patterns in Iranian adult patients with CD.

According to our findings, there has been a substantial relation between age, BMI, and total energy intake with quartiles of healthy dietary pattern score. In other words, with increasing age, participants had the highest adherence to the healthy dietary pattern while consuming more calories. Accordingly, as expected, higher consumption of calories was associated with a higher BMI in adults. In line with our study, Taetzsch et al. based on a meta-analysis of seven published studies demonstrated that consuming a GF diet was linked to consuming more energy and fat and substantially less dietary fiber [22]. Another cross-sectional study conducted on 130 Iranian adult patients with CD, illustrated that the mean calorie intake in celiac patients was considerably lower compared with non-celiac people [10]. Ciacci et al. also realized that adults with celiac disease who follow a strict GF diet have considerably lower weight, BMI, fat and lean body mass than a healthy control group. It was further offered that the diet of these patients was imbalanced and they consumed a higher percentage of calories from fat and less from carbohydrates [23]. Mazzeo et al. in line with our study, assessed the daily energy of 100 individuals with CD by a 7-day weighed food record and the modified Italian European Prospective Investigation FFQ. Their finding illustrated the calorie intake was 2,144.0 (269.4) and 1,890.7 (535.7), respectively. Also, 16% of individuals were categorized as overweight (BMI ≥ 25) which was in agreement with our finding in terms of BMI [24].

A prospective study also examined how different durations of a GF diet affected the BMI of Iranian CD patients. Patients were divided based on diet adherence periods: under 6 months, 6 months to 2 years, and over 2 years. Women showed significant changes in both body weight and BMI in all three periods whilst on GFD, while men experienced significant differences after more than 2 years on the diet. Those aged 31–60 had notable changes in BMI under 6 months, while adhering for more than 6 months showed changes for individuals aged 18–60. These differences may stem from varied eating habits among age groups and hormonal disparities [25].

In addition, we observed major differences in energy density and some dietary nutrient intake between groups within the same dietary cluster (healthy eating pattern, unhealthy eating pattern and mixed dietary patterns). The reports of a prior research in this regard Mager et al. highlighted that within the same individual diet patterns (including, western diet-supplement users and higher, fat western diet and prudent diet, lower fat) there were some distinct in terms of energy density, glycemic load (GL), fiber and micronutrient intake between youth with CD and mild chronic gastrointestinal controls. For example, children with CD’s diets were characterized by high energy density, higher fiber, GL and selenium intakes versus gastrointestinal complaints [21]. In a recent case-control study of Iranian children and adolescents with CD, significant dietary patterns were identified. It was observed that among CD patients, half were found to exceed their caloric intake, while a majority displayed higher consumption levels of protein, carbohydrate, thiamin, riboflavin, niacin, vitamin B12, and iron compared to the recommended thresholds. Additionally, based on the Recommended Dietary Allowance (RDA), all subjects within the CD group demonstrated inadequate intake of vitamin D, with half experiencing insufficient intakes of vitamins A and E. Although the healthy control group exhibited overall higher nutrient consumption, both groups demonstrated similar quality of diet, underscoring the necessity for dietary enhancements among CD patients. While the intake of macronutrients and select micronutrients exceeded the RDA in both groups, there remains a need to improve the dietary quality specifically tailored to individuals with CD [26]. In contrast, a recent report indicates that less than half of CD patients are meeting the recommended daily fruit intake, both before and during the COVID-19 pandemic [27]. Apparently, the reported controversial results between these studies are likely to be owing to the difference in included population, the method of defining non-adherence, and the method of evaluation of the diet habits and pattern. Nutritious eating hence becomes significantly more critical for patients especially in children and adolescents on highly restrictive diets, such as the GF diet, which typically lacks adequate nutritional quality by nature [27]. Compliance with a GF diet and the maintenance of a high-quality dietary regimen represent pivotal challenges among children and adolescents diagnosed with CD [26].

Based on our results from regression analysis, the highest adherence to a healthy dietary pattern was related to the lowest odd ratio of CD development. Meanwhile, a negative association between the odd ratio of CD incidence and traditional dietary pattern score was found. When we looked at the consumption of the healthy dietary pattern in our study, individuals with CD were consumers of whole grains, nuts, legumes, white meat, other vegetables, and tea and coffee. As regard to available data, the consumption of these foods is the excellent dietary source of fiber, minerals, and vitamins, protein and micronutrients as well as representing healthier food options [10, 22]. Despite being celiac patients, those in our study primarily consumed whole grains such as Sangak, Barbari, Taftoon, and barley, which are widely recognized and commonly consumed as whole grains in Iran. Besides, CD patients with a healthy eating pattern had a limited consumption of fast food, refined grains, sweets and desserts, and snacks. Conversely, adherence to a mixed eating pattern was related to the high consumption of sweets and desserts, refined grains, fast food, red meats as well as a lower intake of starch vegetables and whole grains. These foods are rich in simple sugars and high-glycemic index and fat (especially, saturated fatty acids or cholesterol) and also low in fiber. The study also observed a connection between moderate consumption of meat and beans and reduced levels of beneficial bacteria, specifically Lactobacillus and Firmicutes, in individuals with CD. High bean intake was particularly linked to lower Lactobacillus abundance. These findings suggest that the consumption of meat and beans might diminish essential gut bacteria, potentially influencing dietary effects and the continuation of CD symptoms through changes in gut microbiota abundance [28]. As a result, this dietary profile could also be harmful and cause the aforementioned insulin resistance, hyperinsulinemia, and cardiovascular disease [20]. A recent study in this field presented that the lower intake of whole grains, plant protein, legumes, and refined grain in Iranian CD patients than that of non-celiac people controls [10].

However, we should note that Iranian celiac patients have insufficient access to available commercial gluten-free alternative sources knowledge regarding adding healthy foods to the GF diet needs to be promoted. Apart from this, unappealing and unacceptability to some patients and the higher cost of a substitute non-gluten source are other reasons not to choose them [10]. Another case-control study displayed that the participants with CD consumed a substantially lower mean daily energy, carbohydrates (CHOs), total protein, and vegetable protein than that of healthy individuals, while the intake of red and processed meat and lipids was significantly higher in individuals with CD versus healthy participants. According to typical Mediterranean foods, their researchers observed that individuals with CD had a lower intake of fruits as compared to healthy participants, while both groups also ate other typical Mediterranean foods in low amounts. As a consequence, a low adherence to a Mediterranean diet contributed to a remarkable increase in the risk of all-cause, non-communicable diseases mortality [29]. In a study conducted on children with CD, Mager identified three diet patterns reflecting a typical Western Diet pattern (characterized by higher fat/simple sugar intakes) and a more prudent dietary pattern (Lower Fat/Higher CHO diet). None of the patterns reflected a Dietary Approaches to Stop Hypertension (DASH) or Mediterranean diet pattern and also addressed no relation between diet quality by dietary pattern method and adherence to the GF Diet [21]. Whereas, a recent cross-sectional questionnaire-based study involving 37 Dutch pediatric patients diagnosed with CD revealed that the majority of children with CD tended to adhere to a healthier diet. Although, there is a need to address potential unhealthy eating habits that might have developed in these patients [27].

While, the findings of the present study should be interpreted cautiously for some limitations. because food groups and dietary intakes were evaluated using FFQ, thus its limitations such as recall bias and underreporting by participants may have affected the present results. Besides, celiac patients are aware of being health conscious. However, the FFQ was validated in the general Iranian population and modified to examine the dietary habits of patients with CD and accomplished by a trained nutritionist. Another limitation was related to the gastrointestinal symptoms caused by celiac individuals were not assessed which might have had effects on food groups and nutrient intake. Our observation showed that dairy products and fruits and vegetables were not reported in healthy and unhealthy eating patterns. Our assuming that celiac individuals tend to decrease the consumption of these types of food groups resulting in lactose intolerance and non-digestible carbohydrates containing fruit and vegetables consumed that could lead to GI discomforts among patients [10]. Which also might have had minimal effects on our results.

## Conclusion

The main finding of our study, along with a review of the literature, revealed that the patients who consumed healthy dietary patterns reflective of typical DASH or Mediterranean diet patterns had a lower odds ratio of celiac disease incidence. In this matter, our finding proposes that an adequate nutritional educational program and healthcare policies are needed to promote the degree of knowledge or motivation of celiac patients, thus helping patients strictly adhere to the GFD and enhance the nutritional quality of this type of diet, which would be the key to a long-term balanced diet.

### Electronic supplementary material

Below is the link to the electronic supplementary material.

Supplementary Material 1

## Data Availability

The data that support the findings of this study are available from the corresponding author upon reasonable request.

## Abbreviations

CD: Celiac disease
GFD: Gluten-free diet
BMI: Body mass index
FFQ: Food frequency questionnaire
PCA: Principal component analysis
GL: Glycemic load
RDA: Recommended Dietary Allowance
CHOs: Carbohydrates
DASH: Dietary Approaches to Stop Hypertension
SFA: Saturated fatty acid
MUFA: Monounsaturated fatty acid
PUFA: Polyunsaturated fatty acid
TFA: Trans fatty acid
