# Supplementary material for: Assessment of dietary patterns in celiac disease patients using factor analysis method and their relationship with dietary intakes and body mass index
Source: BMC Nutr. 2024 Mar 6;10:48. doi: 10.1186/s40795-024-00849-7 (PMC10916276; doi:10.1186/s40795-024-00849-7)

Supplementary Table 1. Food groups used in the factor analysis

| Food groups | Food items |
| --- | --- |
| Whole grains | Dark breads (Sangak, Barbari, Taftoon), barley |
| Refined grains | White breads (Lavash, Baguette), noodles, pasta, white rice |
| Red meats | Beef, lamb, organ meats |
| White meats | Chicken with or without skin, Canned tuna fish, other fish |
| Eggs | Eggs |
| Legumes | Beans, peas, broad beans, lentils, soy, mung bean, split pea |
| Dairy products | Milk (skimmed, low-fat, high-fat, whole), yoghurt (low-fat, high-fat, cream), Dough (yogurt drink), cream cheese, other cheeses, chocolate milk, ice cream, kashk (drained yogurt product) |
| Fruits and juices | Pears, apricots, cherries, apples, raisins or grapes, bananas, cantaloupe, watermelon, oranges, grapefruit, kiwi, grapefruits, strawberries, peaches, nectarine, tangerine, mulberry, plums, persimmons, pomegranates, lime, fresh figs and dates, Dried figs, dried dates, dried mulberries and other dried fruits, apple juice, orange juice, cantaloupe juice, other fruit juice, fruit compote |
| Starchy vegetables | Potatoes, green peas, pumpkin, turnip, corn |
| Other vegetables | Cabbage, cauliflower; Brussels sprouts, kale, Carrots, Tomatoes, Cucumber, eggplant, celery, green peas, green beans, green pepper, mushrooms, onions, garlic, spinach, lettuce, lemon, summer squash, mixed vegetables |
| Nuts | Peanuts, almonds, pistachios, hazel-nuts, roasted seeds, walnuts |
| Fats and oil | Hydrogenated fats, animal fats, mayonnaise, butter, margarine, cream, vegetables oils, olives and olive oils |
| Fast food | Sausages, hamburger, lunch meat, pizza |
| Snacks | Potato chips, Cheezie, crackers, French fries |
| Tea and coffee | Tea, coffee |
| Sweets and desserts | Chocolates, cookies, cakes, confections, biscuits, halva |
| Sugars | Sugars, candies, gaz, sohan (kind of confectionery), jam, honey, cola |
| Spices and flavors | Tomato sauce, pickles, salt |

Supplementary Table 2. Factor loadings of food groups in major dietary patterns^1^

| Food groups | Major dietary patterns | | |
| --- | --- | --- | --- |
|  | Healthy | Unhealthy | Traditional |
| Snack | -0.59 | 0.43 |  |
| White meat | 0.58 | 0.28 |  |
| Sweets and desserts | -0.53 |  | 0.21 |
| Other vegetables | 0.50 |  | 0.30 |
| Legumes | 0.47 |  |  |
| Refined grains | -0.46 |  | 0.20 |
| Tea and coffee | 0.43 |  |  |
| Starchy vegetables |  | 0.80 |  |
| Sugars |  | 0.75 |  |
| Fats and oils |  | -0.41 |  |
| Nuts | 0.24 | 0.38 |  |
| Spices and flavors | -0.29 | 0.32 |  |
| Fruits and vegetables |  |  | 0.70 |
| Whole grains | 0.43 | -0.32 | -0.63 |
| Eggs |  |  | 0.52 |
| Fast food | -0.35 |  | 0.47 |
| Dairy products |  |  | 0.37 |
| Red meats |  |  | 0.35 |

^1^Factor loadings of less than 0.2 have been omitted for simplicity

Supplementary Figure 1. Scree plot from principal components analysis in the full sample


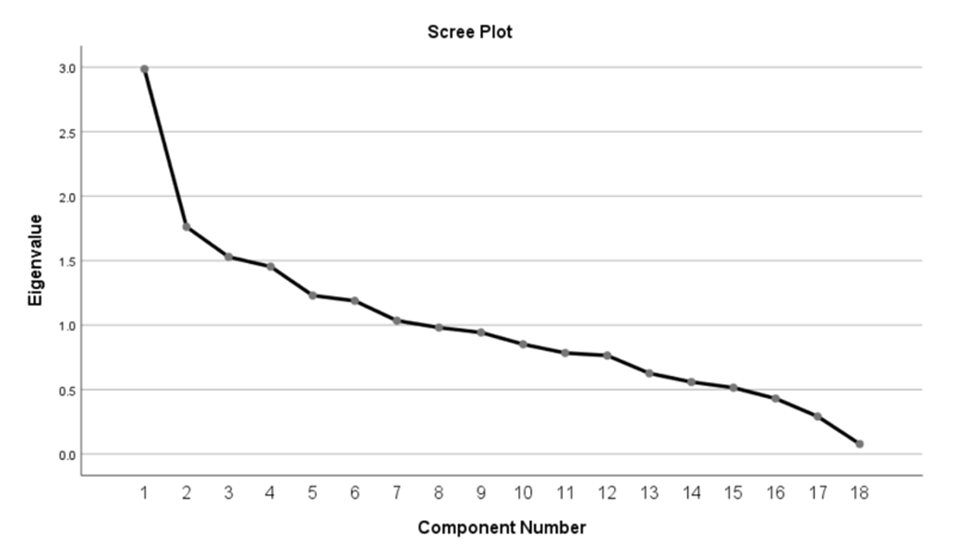

Supplement: Supplementary file 1 — Supplementary Material 1 [file 40795_2024_849_MOESM1_ESM.docx]
